# How social cognition can inform social decision making

**DOI:** 10.3389/fnins.2013.00259

**Published:** 2013-12-25

**Authors:** Victoria K. Lee, Lasana T. Harris

**Affiliations:** ^1^Department of Psychology and Neuroscience, Duke UniversityDurham, NC, USA; ^2^Center for Cognitive Neuroscience, Duke UniversityDurham, NC, USA

**Keywords:** social cognition, person perception, social decision-making, economic games, computers

## Abstract

Social decision-making is often complex, requiring the decision-maker to make inferences of others' mental states in addition to engaging traditional decision-making processes like valuation and reward processing. A growing body of research in neuroeconomics has examined decision-making involving social and non-social stimuli to explore activity in brain regions such as the striatum and prefrontal cortex, largely ignoring the power of the social context. Perhaps more complex processes may influence decision-making in social vs. non-social contexts. Years of social psychology and social neuroscience research have documented a multitude of processes (e.g., mental state inferences, impression formation, spontaneous trait inferences) that occur upon viewing another person. These processes rely on a network of brain regions including medial prefrontal cortex (MPFC), superior temporal sulcus (STS), temporal parietal junction, and precuneus among others. Undoubtedly, these social cognition processes affect social decision-making since mental state inferences occur spontaneously and automatically. Few studies have looked at how these social inference processes affect decision-making in a social context despite the capability of these inferences to serve as predictions that can guide future decision-making. Here we review and integrate the person perception and decision-making literatures to understand how social cognition can inform the study of social decision-making in a way that is consistent with both literatures. We identify gaps in both literatures—while behavioral economics largely ignores social processes that spontaneously occur upon viewing another person, social psychology has largely failed to talk about the implications of social cognition processes in an economic decision-making context—and examine the benefits of integrating social psychological theory with behavioral economic theory.

What makes social decision-making unique and different from non-social decision-making? Humans are highly social animals—as such, researchers often take for granted the ease with which humans make social decisions. This begs the question whether social decision-making is a simplified type of decision-making. Yet social decision-making should be a complex process—social decision-makers must engage traditional decision-making processes (e.g., learning, valuation, and feedback processing), as well as infer the mental states of another person. These two tasks have been separately studied in the fields of behavioral economics and social psychology, with behavioral economists studying decision-making in interactive economic games and social psychologists studying spontaneous inferences about other people. Each of these fields has separately made major contributions to the understanding of social behavior. However, a more cohesive theory of social decision-making results when researchers combine these literatures.

When talking about social decision-making, many different types of decisions may come to mind—decisions about other people (Is Linda a feminist bank teller?), decisions that are influenced by other people (e.g., social conformity and expert advice), as well as decisions that are interactive (e.g., two people want to go to dinner but have to decide on a restaurant). In this review, we focus on strategic interaction decisions often employed in behavioral economics games (e.g., trust game, ultimatum game, prisoner's dilemma game, etc.) that require thinking about the mental states of another person. Research shows that such decisions may differ depending on whether the interaction partner is another person or a computer agent. Here, we suggest that such differences in decision-making arise due to differences when processing human and computer agents. Specifically, viewing another person engages the social cognition brain network, allowing for mental state inferences that function as predictions during the decision phase, as well as spontaneous trait inferences that occur when viewing the other person's behavior in the feedback phase.

To understand how decision-making in a social context is different than non-social decision-making, it is first important to understand what exactly makes humans unique as social agents. Social psychological theory suggests humans differ from objects in important ways (Fiske and Taylor, 2013). First, humans are intentional agents that influence and try to control the environment for their own purposes. Computers on the other hand are non-intentional agents. The decisions made by a computer result from fixed, preprogrammed algorithms, and are usually not as flexible as human decision-making. Second, people form impressions of others at the same time others are forming impressions of them. Therefore, in a social situation people are trying to form impressions of another person at the same time they are trying to manage the impression being formed of them. In meaningful social interaction (most social interactions) the first person usually cares about the reputation the second person is forming of them, wanting them to form a largely positively valenced impression. Each interaction partner is aware that they are the target of someone's attention and may monitor or change their behavior as a result. Third, it is harder to verify the accuracy of one's cognitions about a person than they are about an object. Because things like traits, which are essential to thinking about people, are invisible features of a person and are often inferred, it is harder to verify that a person is trustworthy than it is to verify that a computer, for example, is trustworthy. This may be because the person can manipulate trait information such as trustworthiness—an immoral person can act in moral ways when desired—but a computer has no such desire. Last, and perhaps most importantly, humans possess mental states—thoughts and feelings that presumably cause behavior—that are only known to them. People automatically try to infer the mental states of others because such inferences facilitate social interactions. Computers, however, do not have mental states because they do not have minds. This important distinction—the possession of mental states—allows for the differences mentioned above in intentionality and impression management. These key differences allow us to examine what these social cognitive processes (impression management and intentionality) contribute to the uniqueness of social decision-making, though this discussion seems to often elude studies of social decision-making.

There are also important similarities between humans and computers that make computers the ideal comparison in social decision-making studies. With analogies comparing the human brain to a computer, it almost seems natural that many studies have turned to computers as the non-social comparison. Computers, like humans, are *agents* that can take actions toward a participant. Presumably a computer can “decide” to share money in a trust game as can a human partner. Additionally both humans and computers are information processing systems. Participants' decisions are presumably “registered” by both human and computer agents. Advanced computer programs can take participants' choices into account in order to “learn” to predict another person's behavior using programmed algorithms. For example, website ads learn to predict what a person may purchase based on search history. In some economic games, a computer's responses may be dependent on the participant's past decisions. These similarities allow researchers to compare decisions across agents and examine what social agents add to the decision-making process.

## Social decision-making brain regions

One way to understand the unique nature of social decision-making is to take a neuroscientific approach. By understanding what goes on in the brain, we can begin to dissociate social and non-social decisions. This strategy is particularly informative and useful because similar behavior is sometimes observed for social and non-social stimuli, but the neural mechanisms underlying those decisions are found to be different (e.g., Harris et al., 2005; Harris and Fiske, 2008). Below, we briefly summarize two brain networks we believe will be involved in social decision-making—the traditional decision-making brain network, and the social cognition/person perception brain network^1^. As a caveat, the reader must remember when discussing the unique qualities of social decision-making, we are still examining decision-making. As such, traditional decision-making processes and brain structures underlying these processes are involved in social decision-making studies. Past studies demonstrate that the social context modulates these decision-making structures (see Engelmann and Hein, 2013 for review). However, exactly *how* the social context does this is not entirely understood. By looking in the social cognition/person perception brain network, researchers are beginning to explore how these functions are integrated at a neural level (e.g., Hampton et al., 2008; Yoshida et al., 2010; Suzuki et al., 2012). Next, we list brain regions implicated in decision-making and social cognition.

Past research shows decision-making brain regions are also involved in social decision-making. The medial prefrontal cortex (MPFC)—responsible for creating value signals for food, non-food consumables, and monetary gambles (Chib et al., 2009)—is also active when creating value signals in a social context (Lin et al., 2012). These value signals can be thought of as a quantifiable signal for making predictions—those assigned a higher value predict a better outcome, and those assigned a lower value predict a worse outcome. Recently, it has been suggested that the MPFC works as an action-outcome predictor concerned with learning and predicting the likelihood of outcomes associated with actions (Alexander and Brown, 2011). Similarly, investigations of social reward processing suggest that the striatum responds to both social and monetary rewards (Izuma et al., 2008, 2010). The connections between cortical and subcortical regions with the striatum create a network of brain regions engaged during decision-making. The neurotransmitter dopamine provides a vehicle by which these brain regions communicate. Prediction error signals—the firing of dopamine neurons when observed outcomes differ from expectations (or predictions)—also occur for social stimuli in economic games (Lee, 2008; Rilling and Sanfey, 2011) as well as when social targets violate expectations (Harris and Fiske, 2010). Collectively these regions, along with other regions such as the amygdala, posterior cingulate cortex (PCC), insula, and other areas of prefrontal cortex including orbital prefrontal cortex and a more rostral region of MPFC make up a decision-making network often engaged during economic decision-making (Knutson and Cooper, 2005; Delgado et al., 2007).

While social decision-making studies have investigated how the striatum and prefrontal cortex are modulated by the social context, another prevalent question is whether a network of brain regions established in the social neuroscience literature on social cognition and person perception is also active during social decision-making and how these brain regions interact. An important part of social cognition consists of inferring mental states, like the intentions of a social target (Frith and Frith, 2001). During tasks that involve dispositional attributions—an inference of an enduring mental state—areas such as MPFC and superior temporal sulcus (STS) are reliably activated (Harris et al., 2005). Other areas involved in person perception include temporal-parietal junction (TPJ), pregenual anterior cingulate cortex (pACC), amygdala, insula, fusiform gyrus of temporal cortex (FFA), precuneus, posterior cingulate, temporal pole, and inferior parietal cortex (IPL; Gallese et al., 2004; Haxby et al., 2004; Amodio and Frith, 2006). Together these regions represent a social cognition network that can be used to navigate the social world. This network is believed to be activated in a variety of social cognition tasks, including thinking about others' intentions and goals (i.e., theory of mental state tasks), identifying social others (i.e., faces and bodily movement), moral judgments, social scripts, and making trait inferences (see Van Overwalle, 2009, for a review). However, until recently the mention of these regions in social decision-making studies has been scarce, often being relegated to a supplemental analysis or table. Presumably these social cognitive processes are relevant for decision-making when interacting with human agents because they occur automatically and with minimal exposure to the social target (Ambady and Rosenthal, 1992; Willis and Todorov, 2006). Therefore, these automatic social processes are most likely engaged in a social decision-making context and perhaps provide the vehicle through which the social context modulates decision-making brain regions like the striatum and PFC.

## Differences in social and nonsocial decision-making processes

**Figure 1:**
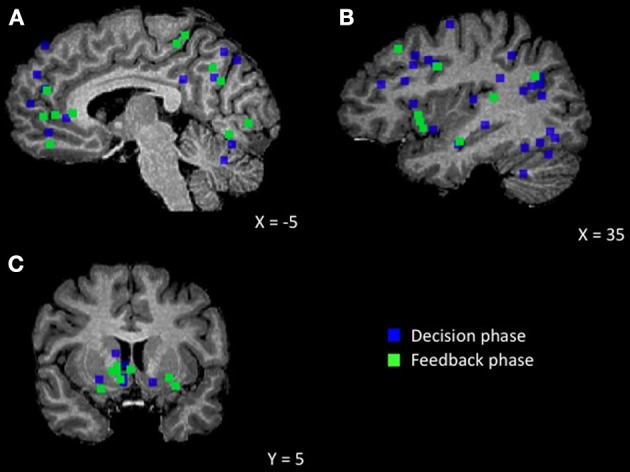
**Brain regions showing an effect of human agent compared to non-social control. (A)** Medial view displaying MPFC, posterior cingulate/precuneus, cerebellum **(B)** Lateral view displaying STS, TPJ, DLPFC, IPL, insula, fusiform **(C)** Coronal view displaying striatum.

Decision-making in its most basic form can be broken down into three key processes^2^ , (1) making predictions that guide decision-making, (2) examining the outcome of the decision, and (3) using the outcome to update predictions, a process often described as learning. Next, we discuss differences between humans and computers for each of these aspects of decision-making to understand how social decision-making is unique (see Figure 1 for a summary of these findings).

### Social predictions

Predictions have received much attention when studying social decision-making. Behavioral economics games such as the trust game, ultimatum game, or the prisoner's dilemma game are often used to study social preferences for trustworthiness, fairness, or cooperation, respectively. However, each of these games requires *predicting* what another agent (person or computer) will do. The combination of the participant's and the partner's decisions determines the outcome. Therefore, in order to maximize payout, the participant has to predict what the partner will do and decide accordingly. What information do participants rely on when making these predictions? Social psychological theory suggests these predictions rely on trait inferences that occur when viewing the person and learning about their past behavior, while also taking the social context into account. Yet discussions of how these predictions are utilized within a decision-making context have eluded social psychology researchers in favor of understanding the processes by which such predictions are made. Below, we discuss these social cognitive processes and how they influence social decision-making in various behavioral economic paradigms involving human and computer agents.

Social decisions are not made within a vacuum; they are made in a social context. A social context involves the actual, imagined, or implied presence of another person—an intentional agent—whose behavior cannot be predicted with certainty. Although humans have developed ways to try to predict what another person will probably do, the other person has the ability to originate their own actions and only they know their true intentions. Therefore, social decision-making is complicated by the uncertainty of the other person's behavior and requires inferences about a person's mental state. Despite these uncertainties, humans are highly motivated to explain and predict others behavior (Heider, 1958). To facilitate this process, humans have developed skills to automatically assess or infer certain types of social information about another person that will guide predictions about their behavior. The primary dimensions of person perception—trait warmth and trait competence—allow for these predictions (Asch, 1946; Rosenberg et al., 1968; Fiske et al., 2007). While trait warmth describes a person's good or bad intentions, trait competence describes the person's ability to carry out those intentions. Research suggests that although these two traits are often assessed together (Fiske et al., 2002), trait warmth carries more weight when forming impressions (Asch, 1946). As such, it is not surprising that the majority of social decision-making studies have capitalized on participants' ability to infer something about warmth-related constructs, including trustworthiness, fairness, and altruism in economic games.

But social predictions are not always formed based on trait inferences alone—social category information (e.g., age, race, gender) and physical features (e.g., facial trustworthiness, attractiveness) can guide initial impressions of a person as well (Fiske, 1998; Ito and Urland, 2003; Ito et al., 2004). Stereotypes—schemas about how people belonging to social categories behave—can act as heuristics for predicting a person's behavior based on this category information (Fiske, 1998; Frith and Frith, 2006). However, these predictions can often be misleading because they do not require mental state inferences for the individual person. Despite this, social category information such as gender and race affect social decisions in an economic context (Slonim and Guillen, 2010; Stanley et al., 2011), suggesting this social information is incorporated into the decision-making process when interacting with human agents.

The basis of these social predictions (e.g., social category information, physical features, and trait inferences) are often assessed automatically and efficiently, with only 100 ms of exposure to a person's face leading to accurate assessments (Willis and Todorov, 2006). These initial impressions may be further supported or adjusted based on the person's behavior. People spontaneously attribute traits to a person based on brief, single acts (thin slices) of behavior. When exposure time to a person's behavior is increased from 30 s to 4 to 5 min, predictions about their future behavior are just as accurate as with minimal exposure (Ambady and Rosenthal, 1992). Therefore, these automatic social processes may influence any social decision-making study that has an actual, imagined, or implied presence of another person.

The development of attribution theory (Heider, 1958; Kelley, 1972; Jones, 1979) further suggests that people are highly motivated to predict and explain behavior and are able to do so quite efficiently. Kelley (1972) suggests only three pieces of information—what other people do (consensus), reliability of a behavior across contexts (distinctiveness), and reliability of a behavior across time (consistency)—are needed for participants to form enduring trait inferences and attribute behavior to a person rather than the situation. Specific combinations—low consensus, low distinctiveness, and high consistency—lead participants to attribute behavior to the agent (McArthur, 1972). Interestingly, research shows that this attribution process may be different for social and non-social stimuli. When this paradigm was taken to the scanner, Harris et al. (2005) showed that attributions for human agents rely on a distinct set of brain regions, including MPFC and STS. However, when the agents are anthropomorphized objects, the same combination of statistical information led to attributions (i.e., the same behavior for human and objects) but a different pattern of brain activity resulted (Harris and Fiske, 2008). Specifically attributions for objects did not engage MPFC but rather STS and bilateral amygdala. These studies, in combination with studies showing increased activity in dorsal regions of MPFC for people compared to objects (cars and computers) in an impression formation task (Mitchell et al., 2005) suggest separable brain systems for people and objects and provide a first hint toward what makes social decision-making different.

What does social psychology teach us about social decision-making studies? Participants use a variety of heuristics that allow them to infer traits and mental states about another person. Whether this is information about their identity (e.g., age, race, gender) or information about their past behavior, participants are constantly trying to make predictions about what other people will do (even outside of a decision-making context). As such, traits provide a concise schema suggesting how a person will behave, allowing for generalizations across contexts when making predictions about behavior. In general, if a person is thought to be trustworthy in one context, people predict that they will be trustworthy in other contexts. Whether actual consistency across contexts exists depends on the psychological viewpoint one takes—personality psychologists would suggest traits are an enduring quality that stays consistent across situations, however, social psychologists stress the importance of the situation and the interaction between person and environment (Lewin, 1951; Ross and Nisbett, 1991).

How does this contribute to our discussion of human and computer agents in an economic game? Do participants use the same brain regions when making predictions about what a human will do vs. what a computer will do? Since each type of agent recruits different brain regions, do social predictions rely on the person perception/social cognition network as we hypothesize above? Below we describe three economic games—the trust game, ultimatum game, and prisoner's dilemma game—often used in the neuroeconomics literature on social decision-making and discuss how social cognition and social psychological theory may be useful when studying these games. We also review research that will help us understand the brain regions underlying these predictions, specifically studies that use non-social agents (e.g., computers) as a control and examine activation during the decision phase when participants are making predictions about what the other agent will do (see Table 1 for list of studies).

**Table 1:** **Summary of studies comparing human and non-social agents**.

**Phase**	**Author**	**Method**	**Task**	**Nonsocial comparison**	**Brain regions associated with an effect of human agent**
Decision	McCabe et al., 2001	fMRI	Trust game	Computer	MPFC
Decision	Gallagher et al., 2002	PET	Rock-Paper-Scissors	Computer	pACC
Decision	Singer et al., 2004	fMRI	PDG	Nonintentional human	fusiform gyrus, STS, insula, vSTR, OFC
Decision	De Quervain et al., 2004	fMRI	Punishing defector in trust game	Random device	caudate nucleus
Decision	Rilling et al., 2004a	fMRI	UG andPDG	Computer	DLPFC, STG, fusiform gyrus, precentral gyrus, inferior frontal gyrus, superior frontal gyrus, posterior cingulate, frontal pole, caudate, cerebellum
Decision	Delgado et al., 2005	fMRI	Trust game	Lottery	IPL, insular cortex, lingual gyrus, putamen, inferior occipital gyrus, vSTR, fusiform gyrus
Decision	Knoch et al., 2006	fMRI	UG	Computer	DLPFC
Decision	Krach et al., 2008	fMRI	PDG	Anthropomorphized robot, functional robot, computer	MPFC, TPJ
Decision	Coricelli and Nagel, 2009	fMRI	Beauty contest	Computer	MPFC, rACC, STS, PCC, TPJ
Decision	Burke et al., 2010	fMRI	Purchasing stocks	Chimpanzees	vSTR
Decision	Carter et al., 2012	fMRI	Poker game/bluffing decisions	Computer	TPJ
Decision	Delgado et al., 2008	fMRI	Auction	Lottery controlled by computer	precuneus, inferior parietal lobe
Feedback	Rilling et al., 2002	fMRI	PDG	Computer	paracentral lobule, caudate, postcentral gyrus, medial frontal gyrus, rostral anterior cingulate gyrus, superior temporal gyrus, paracentral lobule
Feedback	Sanfey et al., 2003	fMRI	UG	Computer	bilateral insula
Feedback	Rilling et al., 2004b	fMRI	UG and PDG	Computer	STR, VMPFC
Feedback	Rilling et al., 2004a	fMRI	UG andPDG	Computer and Roulette Wheel	STS, hypothalamus/midbrain/thalamus, supierior frontal gyrus, rACC, precuneus, thalamus, hippocampus, putamen
Feedback	Delgado et al., 2005	fMRI	Trust game	Lottery	STR (neutral human)
Feedback	Rilling et al., 2008a	fMRI	PDG	Gamble task	superior temporal gyrus, precentral gyrus, anterior insula, precuneus, lingual gyrus, ACC
Feedback	Delgado et al., 2008	fMRI	Auction	Lottery controlled by computer	STR
Feedback	Phan et al., 2010	fMRI	Trust game	Computer	vSTR
Feedback	Harlé et al., 2012	fMRI	UG	Computer (between group contrast)	anterior insula, OFC, DLPFC, precentral gyrus, superior temporal pole, vMPFC, lateral prefrontal cortex, putamen, SMA, parahippocampal Area, precuneus, ACC, cerebellum, inferior parietal gyrus

Brain regions associated with an effect of human agent (compared to non-social control) include social cognition brain regions. UG, ultimatum game; PDG, prisoner's dilemma game; MPFC, medial prefrontal cortex; pACC, posterior anterior cingulate cortex; STS, superior temporal sulcus; vSTR, ventral striatum; OFC, orbital frontal cortex; DLPFC, dorsolateral prefrontal cortex; STG, superior temporal gyrus; TPJ, temporal parietal junction; PCC, posterior cingulate cortex; SMA, supplemental motor area.

One tool for studying social predictions is the trust game. In a typical trust game scenario, participants have the opportunity to “invest” with or give a sum of money (e.g., $10) to another person. Alternatively, participants can decide to keep the money for themselves and not invest. If the money is given to the partner, it is multiplied by some factor (e.g., tripled to $30) and the partner decides whether or not to share the profit with the investor. If the partner shares with the participant, each receives an equal payout ($15). However, if the partner decides to keep the profit ($30), the participant receives nothing. Participants must predict what the partner will do in order to maximize their payout. If they predict the partner will not share, the participants should not invest and keep the money for themselves. However, if participants predict the partner will share, the participants should invest with the partner, risking the chance that they will lose the whole amount.

How do participants make these predictions if they have never interacted with their partners before? From a social cognition perspective, spontaneous mental state inferences may guide these predictions, resulting in corresponding activity in social cognition brain regions. In fact, research shows that when making such predictions for human and computer agents in a trust game social cognition brain regions including the prefrontal cortex (PFC) and inferior parietal cortex (IPL) are more active for human compared to computer partners when participants decide to invest (McCabe et al., 2001; Delgado et al., 2005). However, no differences are observed in activation when participants do not invest, suggesting that investing in the trust game requires inferring the mental states of the partner.

Past behavior may also inform predictions in the trust game. Remember that people form trait inferences from brief single acts of behavior. In a trust game situation, the partner's decision will allow the participant to infer that the partner is trustworthy (or not) from a single exchange. If this behavior is repeated, the partner will build a reputation (a trait inference) for being trustworthy. When relying on reputation to predict the partner's actions, striatal activation shifts from the feedback phase when processing rewards to the decision phase when viewing pictures of previous cooperators, suggesting that participants are making predictions that previous cooperators will again cooperate in the current trial (King-Casas et al., 2005). Therefore, the striatum is also involved in forming social predictions.

Similarly, participants in the ultimatum game interact with human and computer agents that propose different ways of dividing a sum of money (e.g., $10). While some of these offers are fair ($5 each party), others are unfair ($3 for the participant and $7 for the partner). If the participant decides to accept the offer, the money is divided as proposed. However, if the participant rejects the offer, both parties receive nothing. In an economic sense, any non-zero offer should be accepted in order to maximize payout, especially if partners are not repeated throughout the experiment (one-shot games). However, research suggests that unfair offers are rejected more often when the partner is a human agent than computer agent. Why does the identity of the partner affect decisions if the same economic outcome would result? Perhaps, related to our discussion of flexibility above, participants know that humans respond to the environment and make adaptive decisions. If they see that their unfair offers are being rejected, the participant may predict that the human partner will change their behavior, offering more fair offers. However, a computer may be predicted to propose the same offer regardless of how the participant responds, in which case it would be advantageous to accept any non-zero offer because the participant does not anticipate the computer would respond to his or her rejection of the offers. Rejection may also represent a form of punishment of the partner. If the participant receives a low offer, this suggests that the partner has a negative impression of the participant or is simply a morally bad person (unfair, selfish). Punishment in this light is action against such mental states. However, since computers do not possess mental states, there is no reason to punish them for similar unfair offers.

Research shows that when deciding whether to accept or reject offers proposed by human and computer agents, participants show higher skin conductance responses to unfair offers made by human compared to computer agents (Van't Wout et al., 2006), suggesting increased emotional arousal. The use of repetitive transcranial magnetic stimulation (rTMS) shows disruption of the right dorsolateral prefrontal cortex (DLPFC) leads to higher acceptance rates of unfair offers from human but not computer agents (Knoch et al., 2006). The authors of this study highlight the role of DLPFC in executive control and suggest this region is essential for overriding selfish impulses in order to reject unfair offers. When this region is disrupted, participants are more likely to act selfishly and are less able to resist the economic temptation of accepting any non-zero offer. Although the role of DLPFC in executive control is not debated, a more social psychological explanation may be useful in understanding this behavior as well. Impression management is believed to be part of executive control function (Prabhakaran and Gray, 2012). Therefore, we may ask if DLPFC is involved in overriding selfish impulses specifically or whether concerns about impression management may also be affected by the DLPFC's role in executive control. Accepting and rejecting offers in the ultimatum game communicates something to the partner about the participant—whether or not they will accept unfair treatment. In other words, the participant's behavior allows the partner to (presumably) form an impression of them. In order to manage this impression, participants may reject unfair offers as a way to communicate that he or she will not stand for being treated unfairly. Therefore, perhaps when DLPFC is disrupted with rTMS, impression management concerns are reduced and unfair offers are more often accepted. Concerns about forming a good reputation are also affected by rTMS to right DLPFC in the trust game (Knoch et al., 2009), further suggesting this region may be involved in impression management.

The prisoner's dilemma game (PDG) is another economic game exemplifying the role of predictions in social decision-making. In this game, participants must decide whether to cooperate with a partner for a mediocre reward (e.g., $5 each), or defect in order to receive a better reward at the expense of the partner (e.g., $10 for the participant, $0 for the partner). However, risk is introduced into the game because if the partner also defects, both players end up with the worst possible outcome (e.g., $0). In this case it is important for the participant to predict what the partner will do because the payout structure that both parties receive depends on what each chooses.

When participants believe they are playing with human rather than computer agents, imaging results show greater activation in regions involved in social cognition, including right posterior STS, PCC, DLPFC, fusiform gyrus, frontal pole, along with decision-making regions like the caudate (Rilling et al., 2004a). Time-course data show specifically within posterior STS and PCC there is an increase in activation in response to the human partner's face that remains elevated until the outcome is revealed. This increase in activity in social cognition brain regions to human partners is further supported by a study examining PDG decisions to agents varying in degree of human-likeness. Participants that played the PDG with a human, anthropomorphized robot (human-like shape with human-like hands), functional robot (machine-like shape with machine-like hands), and computer showed a linear increase in MPFC and right TPJ activity as human-likeness increased (Krach et al., 2008).

In addition to the agent's perceived physical likeness to a human, it seems as though the intentionality of the human agents is essential for activating social cognition regions. In a study that manipulated whether human agents were able to decide freely in the PDG (intentional) vs. following a predetermined response sequence (unintentional), Singer et al. (2004) observed increased activation of posterior STS, bilateral fusiform gyrus, bilateral insula, right and left lateral OFC, and ventral striatum for cooperating intentional humans. Therefore, it is not that all humans activate social cognition regions in the PDG, but specifically intentional human agents. Together these studies suggest activity in social cognition brain regions track whether the partner is a social agent and may influence social decisions.

Although these economic games are most often used to study social decision-making, other games also suggest that social cognition brain regions are essential for predicting the actions of others. For instance, when playing a game of Rock-Paper-Scissors with either a human or computer counterpart, Gallagher et al. (2002) observed bilateral activation in pACC for human compared to computer partners. More recently, the TPJ has been identified as providing unique information about decisions involving social agents. Participants playing a poker game with human and computer agents had to predict whether the agent was bluffing. Using MVPA and a social bias measure, Carter et al. (2012) showed that TPJ contains unique signals used for predicting the participant's decision specifically for socially relevant agents but not for computer agents. And lastly, research suggests there are individual differences in the extent to which people use social cognition in a decision-making context. In the beauty contest game, participants must choose a number between 0 and 100 with the aim of choosing a number that is closest to 2/3 times the average of all the numbers chosen by different opponents. When playing this game with human and computer opponents, Coricelli and Nagel (2009) found that human opponents activated regions involved in social cognition, including MPFC, rostral ACC, STS, PCC, and bilateral TPJ. The researchers then examined individual differences in participants' ability to think about others' mental states. While low-level reasoners do not take into account the mental states of others when guessing, high-level reasoners think about the fact that others are thinking about the mental states of others and try to guess accordingly. Interestingly including this individual difference measure in the analysis showed that activity in MPFC was only significant for high-level reasoners.

Together, across different social decision-making paradigms, there seems to be increasing evidence that human and computer agents engage different brain regions when making predictions. Specifically, making predictions about human agents engages brain regions implicated in the social cognition network, including MPFC, STS, TPJ, along with decision-making regions like the striatum. Next we ask whether these social decision-making paradigms engage different brain circuitry when processing feedback from human and computer agents.

## Social feedback

While many studies have suggested that social predictions rely on the social cognition brain network, other social decision-making studies have looked at how the outcome of social decision-making, or social feedback, affects traditional decision-making brain regions involved in reward processing and valuation. Initial attempts to study the uniqueness of social decision-making include examining whether social and non-social rewards are processed in the same areas of the brain, and how economic decisions are made in the context of social constructs including trustworthiness, fairness, altruism, and the like. Using behavioral economic games described above (e.g., trust game, ultimatum game, etc.) researchers have examined the influence of positive and negative feedback on social decisions. Below, we review the results of such studies in an attempt to continue the comparison between human and computer agents in social decision-making.

Social feedback often allows people to infer something about another person as well as receive information about the impression others have formed of them. In the context of receiving direct social feedback about what other people think, research suggests that being labeled trustworthy activates the striatum in much the same way as receiving monetary rewards (Izuma et al., 2008). This concept of trust is important when making decisions in a social context because it affects existing social interactions as well as whether others will interact with you. In the economic trust game described above, feedback about whether or not the partner returns an investment allows for trait inferences about the partner based on thin slices of behavior that may guide future predictions.

When participants play the trust game with another human, reward related regions such as the caudate nucleus are active (King-Casas et al., 2005). With repeated exposure to the partner's behavior, participants form a reputation (an inferred trait) for the partner as being trustworthy or not. When these partners are human and computer agents, participants differentiate cooperating from non-cooperating humans, investing most often with humans that returned the investment, an average amount with a neutral human, and least often with humans that did not return the investment. Investments for the computer agent were similar to the neutral human. Reflecting this pattern of behavior, brain activity within the left and right ventral striatum reveals increased activity to cooperating compared to non-cooperating humans, but activity to computers looks similar to neutral human partners (Phan et al., 2010). These results suggest that if a human agent provides no informative information that allows for a trait inference (a neutral partner is neither good or bad), behavior and brain activity may be similar to that of a computer agent. Similar results are observed when reading descriptions of hypothetical partners' past moral behaviors. When playing the trust game with a neutral investment partner (neither good or bad moral character) activity within the striatum for positive and negative feedback looks similar to when receiving such feedback about a non-social lottery outcome (Delgado et al., 2005). However, when the human agent is associated with a specific moral character, striatal activity for positive and negative feedback look the same, demonstrating that prior social information can bias feedback mechanisms in the brain, but only when the social information is informative about one's traits.

In the trust game, the outcome phase has a clear start and end—participants make a decision to invest (share) with a partner and then receive feedback in the same trial about whether the investment was returned by the partner. However, in the ultimatum game, the outcome phase is less clear—participants already know the outcome of the social interaction when they decide whether to accept or reject the offer made by the agent. However, this does not make the outcome of the social interaction irrelevant. In repeated ultimatum games (when participants play multiple trials with the same partner), feedback about the participant's decision comes on the next trial when the partner proposes the next division of money. For example, if a participant rejects an unfair offer, feedback about whether that rejection was effective in influencing the partner's next proposal comes on the next trial. In other words, offers can be thought of as feedback within the context of this game. However, researchers often use single-shot ultimatum games to avoid effects of repeated interaction just described. In this case, the offers proposed by the partner allow the participant to infer traits about the partner, and their decision still communicates something to the partner, prompting participants to think about impression management.

How then do participants respond to offers made by human and computer agents in the context of the ultimatum game? Research suggests that unfair offers made by human agents activate bilateral anterior insula to a greater extent than the same unfair offers made by computer agents, suggesting that there is something about being mistreated specifically by human agents that leads to higher rejection rates (Sanfey et al., 2003). Additionally it seems as though the balance of activity in two regions—anterior insula and DLPFC—predicts whether offers are accepted or rejected. Unfair offers that are subsequently rejected have greater anterior insula than DLPFC activation, whereas accepted offers exhibit greater DLPFC than anterior insula. Similarly, when viewing a human partner's offer, social cognition and decision-making regions including STS, hypothalamus/midbrain, right superior frontal gyrus (BA8), dorsal MPFC (BA 9, 32), precuneus, and putamen are active (Rilling et al., 2004a). More recent investigations of unfair offers suggest the identity of the agent (human or computer) determines whether mood has an effect on activity in bilateral anterior insula (Harlé et al., 2012). Specifically, sad compared to neutral participants elicited activity in anterior insula and ACC as well as diminished sensitivity in ventral striatum when viewing unfair offers from human agents but there were no such differences for offers made by computer agents. These differences in brain activity for human and computer agents further highlight that social decision-making (compared to non-social) relies on different neural processing.

Unlike the ultimatum game, the prisoner's dilemma game is similar to the trust game, because the participant and the partner must make a decision before finding out the outcome of both parties' decisions. This outcome period lets the participant know whether their predictions about the partner were correct. When participants played the prisoner's dilemma game in the scanner, Rilling et al. (2002) observed different patterns of brain activation during outcome depending on whether the partner was a human or computer agent. Specifically, both human and computer agents activated ventromedial/orbital frontal cortex (BA 11) after a mutually cooperative outcome (both the partner and participant decided to cooperate). However, mutual cooperation with human partners additionally activated rostral anterior cingulate and anteroventral striatum. A few years later, researchers investigated whether these different activations were limited to when partners cooperate. Comparing social to non-social loss (human partners do not cooperate and losing a monetary gamble), Rilling et al. (2008a) observed higher activation in superior temporal gyrus (BA 22), precentral gyrus, anterior insula, precuneus, lingual gyrus, and anterior cingulate for the human agent. This analysis highlights the importance of human agents' perceived intent in the prisoner's dilemma game, as it controls for differences in monetary payoff, frequency, and emotional valence that may have confounded previous comparisons of cooperation and defection. These studies suggest processing outcomes from human and computer agents is different. Specifically, human agents engage social cognition brain regions, perhaps because outcomes lead to spontaneous trait inferences for humans and not computers. This idea is consistent with social neuroscience research showing different activity when attributing behavior to people and objects (Harris et al., 2005; Harris and Fiske, 2008).

In another study, participants played a time estimation task in which a human or computer agent delivered trial-by-trial feedback (juice reward or bitter quinine). Some brain regions, including ventral striatum and paracingulate cortex (PACC) responded more to positive vs. negative feedback irrespective of whether the agent was a human or computer (Van den Bos et al., 2007). Other brain regions, particularly bilateral temporal pole, responded more to feedback from human than computer agents, regardless of feedback valence. However, the combination of type of agent and feedback valence seems to be important within the regions of anterior VMPFC and subgenual cingulate. Interestingly this study is one of the few comparing human and computer feedback that is relevant to the competence rather than warmth domain but delivers the same take home message—some brain regions like the striatum and prefrontal cortex respond to social and non-social stimuli, but others like social cognition regions are engaged specifically to the human agent. Why are social cognition regions engaged if feedback was dependent on the participant's performance in the task and not the agents' decisions (i.e., delivered feedback did not allow for a trait inference about the agent)? It may be that participants were concerned about the impression the human agent formed of them (i.e., participants know their behavior allows for trait inferences about them in the same way they form trait inferences about others), but these concerns were not relevant for the computer agent because computers do not form impressions.

Another study examining the effects of competing against a human or computer in an auction suggests that differences in brain activity during outcome depend on both the type of agent and the context of the outcome (Delgado et al., 2008). Participants were told that they would be bidding in an auction against another human or playing a lottery game against a computer and had the opportunity to win money or points at the end of the experiment. The points contributed to the participant's standing at the end of the experiment in which all participants would be compared. In other words, the points represented a social reward, allowing participants to gain status when comparing themselves to other participants in the study. In both cases the goal was to choose a number higher than that chosen by the other agent. When the outcome of the bidding was revealed, the authors observed differential activity for the social and lottery trials. Specifically, losing the auction in the social condition reduced striatal activity relative to baseline and the lottery game. The authors suggest that one possible explanation for overbidding in auctions is the fear of losing a social competition, which motivates bids that are too high, independent from pure loss aversion. These differences for social and non-social loss highlight again that although the same brain regions are active, the social context modulates activity within decision-making regions.

But should we be surprised that social loss seems more salient to participants in a social competition such as the one created by the experimenters? Specifically, the experimenters told participants that final results about the participant's standing in relation to other participants would anonymously be released at the end of the study in a list of “Top 10 players.” Even though there was no risk of identifying a particular participant, social concerns about impression management may have still been active. Being listed as one of the top players allows the trait inference of being very competent in the auction, a desirable trait to almost anyone. Therefore, participants may have believed that negative feedback (losing the auction trials) would lead people to infer that they were inferior or incompetent compared to other players. On the other hand, losses on the lottery trials were simply relevant to the participants and not their social standing.

Converging evidence suggests that common brain regions, particularly the striatum and VMPFC, are engaged when viewing outcomes from human and computer agents. However, the activity in these regions seems to be modulated by the social context. In addition to these decision-making regions, the ultimatum game and prisoner's dilemma game also activate regions involved in social cognition, including STS, precuneus, and TPJ. Should it be surprising that social cognition regions are also active during outcomes? Social psychology demonstrates that people infer traits from others' behavior. The outcome of a social interaction allows participants to infer these traits, and what perhaps is even more interesting is that these trait inferences are formed in single-shot games where participants do not interact with the partner again. Essentially, trait inferences in this context are superfluous because the participant will not be interacting with the partner again so there is no need to infer traits that allow for predictions. Yet these social cognition regions are still engaged.

## Social learning

So far we have seen that social cognition informs predictions made in social decision-making studies when interacting with human but not (or to a lesser extent) when interacting with computer agents. Social rewards, including being labeled trustworthy by another person (Izuma et al., 2008), gaining social approval by donating money in the presence of others (Izuma et al., 2010), and viewing smiling faces (Lin et al., 2012) engage brain regions that are common to receiving non-social rewards, such as money. However, when receiving feedback from social and non-social agents, though common brain regions including the striatum are engaged, the type of agent may modulate activity in these regions. Moreover, feedback from a social interaction also engages regions of the social cognition network. Next, we examine differences in social decision-making during the updating or learning process.

Research examining learning in a non-social context has highlighted the role of prediction error signals in learning to predict outcomes. In a now classic study, recordings from dopamine neurons show that primates learn to predict a juice reward, shifting the firing of dopamine neurons to the cue rather than reward. When an expected reward is not received, dopamine neurons decrease their firing (Schultz et al., 1997). Similar prediction error signals have been observed to social stimuli in both an attribution task (Harris and Fiske, 2010) as well as in decision-making contexts (King-Casas et al., 2005; Rilling et al., 2008b for review). In recent years, it has therefore been suggested that social learning is akin to basic reinforcement learning (i.e., social learning is similar to non-social learning). When interacting with peers, ventral striatum and OFC seem to track predictions about whether a social agent will give positive social feedback and ACC correlates with modulation of expected value associated with the agents (Jones et al., 2011). It has also been proposed that social information may be acquired using the same associative processes assumed to underlie reward-based learning, but in separate regions of the ACC (Behrens et al., 2008). These signals are believed to combine within MPFC when making a decision, consistent with the idea of a common valuation system (which combines social and non-social) within the brain (Montague and Berns, 2002). In fact, value signals for both social and monetary rewards have been found to rely on MPFC (Smith et al., 2010; Lin et al., 2012) and activity in this region also correlates with the subjective value of donating money to charity (Hare et al., 2010).

However, social learning does not inherently appear to be just another type of reinforcement learning. Social decisions often contradict economic models that attempt to predict social behavior, suggesting that simple reinforcement learning models by themselves are not sufficient to explain complex social behavior (Lee et al., 2005). Research shows that reward and value signals are modulated by the social context. For instance, reward related signals in the striatum are affected by prior social information about an investment partner (Delgado et al., 2005) as well as when sharing rewards with a friend vs. a computer (Fareri et al., 2012). Additionally, research shows that social norms can influence the value assigned to social stimuli, specifically modulating activity in nucleus accumbens and OFC (Zaki et al., 2011). Interestingly, functional connectivity analyses show that value signals in MPFC may rely on information from person perception brain regions like the anterior insula and posterior STS (Hare et al., 2010). Studies investigating how person perception brain regions affect social learning suggest that specific types of social information (warmth vs. competence) affect social learning—whereas information about a person's warmth hinders learning, information about a person's competence seems to produce similar learning rates as when interacting with computer agents (Lee and Harris, under review).

Should we be surprised by findings that social stimuli affect learning and the updating process? Social psychology suggests the answer to this question is no. Behaviorally, people have a number of biases that may affect the way information is processed and incorporated into decision-making processes. Tversky and Kahneman (1974) were perhaps the first to point out these biases and heuristics that may be used in a social decision-making context. For instance, people use probability information to judge how representative a person is of a specific category (representativeness heuristic), and recent events to assess how likely it is that something will occur (availability heuristic). When asked to give an estimate of some quantity, being given a reference point (an anchor) affects the resulting estimates. These heuristics can be applied to a social decision-making context as well. For instance when playing the trust game, participants may use initial impressions formed about the person (based on a representative heuristic about what trustworthy people look like) as an anchor that affects whether or not they invest with the partner on subsequent trials. In addition to this bias, it is harder to verify cognitions about people than objects, making it harder to accurately infer the traits of a person compared to an object (Fiske and Taylor, 2013).

In addition to the heuristics described above, people also possess a number of biases that affect how they interpret information. First, people look for information that is consistent with a preexisting belief. This confirmatory bias is evident in the stereotype literature, which demonstrates that people interpret ambiguous information as consistent with or as a confirmation of a stereotype about a person (Bodenhausen, 1988). This bias is relevant to the economic games employed in social decision-making studies because partners often provide probabilistic (sometimes ambiguous) feedback. Interpretation of this feedback may be influenced by prior beliefs (Delgado et al., 2005). Second, people often exhibit illusionary correlations—that is they see a relationship between two things when one does not exist (Hamilton and Gifford, 1976)—and are more likely to attribute a person's behavior to the person rather than to some situational factor (Jones and Davis, 1965; Jones and Harris, 1967; Ross, 1977; Nisbett and Ross, 1980). This again leads participants in social decision-making studies more likely to interpret a partner's decision as a signal of some underlying mental state or trait attribute rather than positive or negative feedback in a purely reward processing sense.

How then can we reconcile these two different literatures, one stating that social learning is similar to reinforcement learning, and another stating that social learning includes a number of biases? In more practical terms, we know that impressions of a person can guide decision-making. Previous studies have shown that facial trustworthiness affects investment amounts in the trust game (Van't Wout and Sanfey, 2008). However, first impressions are not the only influence on social decisions—if someone is perceived as trustworthy that does not make their subsequent behavior irrelevant. Other research has shown the importance of prior behavior on trust decisions (Delgado et al., 2005; King-Casas et al., 2005). To study how the combination of impressions and behavior affect social decision-making, Chang et al. (2010) used mathematical models based on reinforcement learning to test specific hypotheses about how these two types of information guide social decisions in a repeated trust game. Specifically, the authors tested three models that suggest different ways of processing information and investigate whether reinforcement learning or social biases influence decision-making. First, an Initialization model assumes that initial impressions (implicit trustworthiness judgments) influence decision-making at the beginning of the trust game, but eventually participants learn to rely on the player's actual behavior. A Confirmation Bias model assumes that initial impressions of trustworthiness affect the way feedback is processed, the impression is updated throughout the study, and learning is biased in the direction of the initial impression. The third, Dynamic Belief model, assumes that initial impressions are continuously updated based on the participant's experiences in the trust game and these beliefs then influence learning. In this model, equal emphasis is placed on the initial judgment and the participant's experience. That is, initial trustworthiness is simultaneously influencing learning and being updated by experience. Of the three models, the Dynamic Belief model fit the data the best, suggesting that both social cognition processes (initial impressions) and decision-making processes (feedback processing) affect social learning in the trust game.

More recent social decision-making studies have investigated how social processes affect learning. Researchers have proposed different strategies participants may use when learning to predict what their partner will do. One such strategy is learning to simulate other people's decisions and update those simulations once the other's choice is revealed. This process engages different regions of prefrontal cortex involved in valuation and prediction error (Suzuki et al., 2012). Another strategy is to account for the influence one's decisions have on the partner's decisions and decide accordingly. This strategy requires predicting how much influence one has on the partner and updating that influence signal when observing the partner's decision. Computational modeling suggests MPFC tracks the predicted reward given the amount of expected influence the participant's choices have on the partner, and STS activity is responsible for updating the influence signal (Hampton et al., 2008). Although these studies do not provide direct comparisons to non-social controls, they provide exciting insight into how social cognition processes affect social learning.

## Conclusion

Is social decision-making unique? How does it differ from non-social decision-making? The answers to these questions have been of interest to researchers in a variety of fields including social psychology and behavioral economics. Combining these literatures can help us understand the answers to these questions. Economists originally believed that social decision-making was not different from non-social decision-making and tried to model social decisions with traditional economic models. However, after the influential paper by Tversky and Kahneman (1974) demonstrating heuristics and biases affecting decision-making, it became apparent that the decision-making process is not as rational as we may have originally thought. Psychologists have long believed that social cognition is important for predicting the actions of others and that humans are different from objects in some very important ways. More recently, brain-imaging studies have highlighted these differences, with a network of brain regions responding to social stimuli and social cognitive processes that presumably affect social decision-making. Investigations of social decisions have also highlighted the effects of social information on decision-making processes within brain regions like the striatum and MPFC. Although both social and non-social agents engage these brain regions, the social context modulates this activity. The use of mathematical models suggests that both social neuroscience and neuroeconomics studies have each been tapping into different processes. Initial impressions allow for predictions that guide decision-making. These impressions then interact with feedback processing and affect how predictions are updated.

In economics, behavioral game theorists recognize that people's beliefs about others matter when modeling social decisions. The models assume that players strategically choose options that maximize utility, and evaluations of payoff options often include social factors beyond pure economic payout (Camerer, 2009). These social factors may include other-regarding preferences, indicating that people care about the well-being of other players (Fehr, 2009). Whether decisions are made in order to increase the well-being of others or manage the impression formed of oneself, mental state inferences are still relevant. For instance, one may assess well-being by inferring the mental state of the person. Similarly, the extent to which one infers the mental state of a person may influence the extent to which other-regarding preferences influence decisions (e.g., do people show other-regarding preferences for traditionally dehumanized targets?).

Humans evolved in a social context in which interacting with other people was essential for survival. As such, these social cognitive processes have been evolutionarily preserved and continue to affect our decision-making in a social context. The fact that human agents engage different brain regions than computer agents should perhaps not be all that surprising. The social brain did not evolve interacting with computers or other types of machines. Therefore, we see differences not only in behavior (most of the time) but also differences in brain activity for these two inherently different types agents. Here we have highlighted that these differences lie in engagement of the social cognition/person perception brain regions for human agents. But the underlying mechanisms—the social processes that engage these brain regions and how they interact with decision-making processes—are still being investigated. Social psychological theory can help answer these questions by providing a theoretical background for why human and computers differ in the first place (e.g., mental state inferences, impression management, etc). Keeping this fact in mind will provide future research on social decision-making with the most informed and cohesive theories.

Finally, decisions are made in a social context everyday. Whether deciding to do a favor for a friend or close a deal with a potential business partner, decisions have consequences that lead to significant rewards and punishments such as a better relationship with the friend or a poor business transaction. Therefore, it is important to understand how decisions are influenced by the presence or absence of others and how we incorporate social information into our decision-making process. Here we have highlighted differences arising when interacting with human and computer agents and use social psychological theory to provide some explanation for why these differences arise. It is important to point out these differences in social and non-social decision-making because interactions with computers and other machines are becoming more widespread. Businesses often try to find ways to simplify transactions, often replacing human agents with automated computers. However, the decisions made with these different types of agents may affect businesses in unanticipated ways. Financial decisions (e.g., buying and selling stock) are increasingly made through the use of online computers, whereas previously investors had to interact with stockbrokers in an investment firm. Similarly people are able to bid in online auctions for a desired item rather than sitting in a room full of people holding numbered paddles. The decisions to buy and sell stock or possibly overbid in an online auction may be influenced by these different agents, as evidenced by the research described above.

### Conflict of interest statement

The authors declare that the research was conducted in the absence of any commercial or financial relationships that could be construed as a potential conflict of interest.

## Footnotes

^1^However before we begin, it should be noted that it is easy to make these distinctions for discussion purposes here, but each of these processes rely on other brain regions as well and the decision-making process is the result of interactions between these brain regions.

^2^Rangel et al. (2008) suggests five steps for value based decision-making, including the three listed here as well as a representation stage and an action selection stage. We do not focus on these 2 steps here because they may not be all that different for social and nonsocial decision-making.
